# Vibration optimization of spur gear based on GSA-SA algorithm

**DOI:** 10.1371/journal.pone.0293460

**Published:** 2023-11-01

**Authors:** Linyue Qiu, Xiangying Hou, Shushen Gao, Zhengminqing Li, Rupeng Zhu

**Affiliations:** 1 College of Mechanical and Electrical Engineering, Nanjing University of Aeronautics and Astronautics, Nanjing, Jiangsu, China; 2 National Key Laboratory of Helicopter Aeromechanics, Nanjing, Jiangsu, China; National Kaohsiung University of Science and Technology / Industrial University of Ho Chi Minh, TAIWAN

## Abstract

To determine the optimal design parameters of spur gear under a specific condition, based on the basic theories of gear dynamics theory, gear meshing principle, tooth contact analysis and load tooth contact analysis, a six-degree-of-freedom vibration analysis model of spur gear pair is established, and a gravitational search-simulated annealing hybrid algorithm (GSA-SA) is used to optimize the gear addendum modification coefficient and profile modification parameters. The vibration response of the spur gear pair is evaluated through the optimization objective function established by the combination of the G1 method and variation coefficient method. The study shows that the optimized design parameters effectively reduce the level of the vibration, which proves the effectiveness of the optimization method, and the simultaneous optimization of the addendum modification coefficient and profile modification parameters of the gear has a better result than only optimizing the addendum modification coefficient or profile modification parameters. This method can be used for gear transmission system vibration optimization design in the automotive industry and shipbuilding.

## Introduction

Gearing is one of the most commonly used transmission methods, and with the advancement of technology, higher requirements are put forward for the control of transmission system vibration and noise [1–3]. Gears generate shocks and vibrations during meshing due to manufacturing and installation errors, elastic deformation and other factors. In order to reduce the resulting vibration and noise, gear meshing performance is often improved by adjusting the gear addendum modification coefficient and gear profile modification.

There have been many studies on the effect of gear profile modification on gear vibration. Pleguezuelos et al. [4], Tesfahunegn et al. [5], and Sánchez et al. [6] studied the effect of gear profile modification on transmission errors. Pramono [7] experimentally analyzed the effect of gear profile modification on vibration at low speed and torque. Li et al. [8] and Divandari et al. [9] analyzed the effect of gear profile modification on vibration by building finite element models and dynamic models, respectively. Gear addendum modification also helps to suppress gear vibration and noise. Zhao et al. [10] studied the effect of gear addendum modification on gear noise. Baglioni et al. [11] and Yu et al. [12] investigated the effect of displacement coefficient on gear vibration and shock. Some researchers have also conducted gear simulation analysis research based on digital twin [13, 14]. The above researches mainly focuses on optimizing the profile modification parameters or modification coefficients of gears separately, lack of research on the impact of simultaneously optimizing addendum modification coefficient and profile modification parameters on gear vibration reduction.

The limitations of traditional optimization methods mean that they don’t have advantage in solving complex optimization, such as optimization problems without a differentiable objective function. The heuristic algorithms are more suitable for solving optimization problems in engineering [15–22]. The heuristic algorithm does not rely on a priori experience and can quickly search for the optimal solution in the solution space, so it has been introduced and widely used in gear optimization design [23, 24]. Thoan et al. [25] used the algorithm to optimize the gear addendum modification coefficient. Ghosh et al. [26] studied the effect of profile modification parameters on gear vibration and noise by establishing a gear dynamic model; Bonori et al. [27] used genetic algorithms to optimize the static transmission error of gear pairs, with modification parameters as variables, to optimize the parameters of gears to reduce vibration and noise.

In this paper, the design parameter optimization of spur gear pair under a specific condition is studied based on a six-degree-of-freedom spur gear model. The six-degree-of-freedom model is established according to the gear dynamics theory to analyze the vibration response of gears, then the vibration response is weighted to form the optimization objective function by the G1-variation coefficient method. To reduce the vibration and noise of the gear pair the gear addendum modification coefficient and profile modification parameters are taken as the optimization variables, and the GSA-SA hybrid algorithm is used to solve the optimization more efficiently. This optimization method can be applied to the gear optimization design of the automotive industry and shipbuilding, also providing some theoretical basis for the optimization design of the gear transmission system.

## Gravitational search-simulated annealing hybrid algorithm

### Heuristic algorithm

The traditional optimization methods are not very suitable for solving the present optimization problems due to the problems of needing to give differentiable or derivable objective functions or having too much computational complexity of the objective functions. The heuristic algorithm, such as the gravitational search algorithm, is a better choice to solve optimization problems. The gravitational search algorithm has a good global search ability, but it is also easy to fall into the local optimum in the process of convergence [28, 29]. The simulated annealing algorithm has the ability to jump out of local optima, but its global search is easily affected by initial settings [30]. In this paper, the gravitational search algorithm is combined with simulated annealing algorithm to form a gravitational search-simulated annealing hybrid algorithm to improve the algorithm’s ability to solve optimization problems.

### Gravitational search algorithm

The gravitational search algorithm [31] is a heuristic optimization algorithm based on the law of gravity, which has a good global search capability. The gravitational search algorithm treats every agent as an object with mass, and the agents with large mass have more gravitational force on other agents, which causes all the agents in the population to gather toward the large-mass agents, so as to determine the optimal solution to the problem. The basic principle of the gravitational search algorithm is as follows:

Let there be *N* agents in a space and define the agent positions as:

$$X_{i}=(x_{i}^{1},\ldots ,x_{i}^{d},\ldots ,x_{i}^{n})\;\;i=1,2,\ldots ,N$$ (1)

where $x_{i}^{d}$ is the position of the *i*th agent in the *d* dimension.

The force of agent *j* on agent *i* at time *t* is defined as follows:

$$F_{ij}^{d}(t)=G(t)\frac{M_{pi}(t)\times M_{aj}(t)}{R_{ij}(t)+\varepsilon }(x_{j}^{d}(t)-x_{i}^{d}(t))$$ (2)

where *M*_*aj*_ and *M*_*pi*_ are the inertial masses of agent *j* and agent *i*, respectively, and *G(t)* is the gravitational constant at moment *t*,*ε* is a small constant and *R*_*ij*_(*t*) is the Euclidean distance between agent s *i* and *j*:

$$R_{ij}(t)=||X_{i}(t),X_{j}(t)|{|}_{2}$$ (3)

The combined force acting on the *i*th agent in *d* dimensions is a randomly weighted sum of the forces acting on it:

$$F_{i}^{d}(t)=\sum_{j=Kbest,j\neq i}^{N}rand_{j}F_{ij}^{d}(t)$$ (4)

where *rand*_*j*_ is a random number on [0,1].

The position and velocity of agent *i* are:

$$v_{i}^{d}(t+1)=rand_{i}\times v_{i}^{d}(t)+a_{i}^{d}(t)$$ (5)

$$x_{i}^{d}(t+1)=x_{i}^{d}(t)+v_{i}^{d}(t+1)$$ (6)

The masses and inertial masses of the agents are updated by the following functions:

$$m_{i}(t)=\frac{fit_{i}(t)-worst(t)}{best(t)-worst(t)}$$ (7)

$$M_{i}(t)=\frac{m_{i}(t)}{\sum_{j=1}^{N}m_{\text{j}}(t)}$$ (8)

where *fit*_*i*_(*t*) is the fitness value of agent *i* at moment *t*.

The steps of the gravitational search algorithm are as follows: (1) Determine the search space; (2) Randomize initialization population; (3) Calculate the agent fitness values; (4) Update *G(t)*, *best(t)*, *worst(t)* and *M*_*i*_(*t*) ; (5) Calculate the combined forces in different dimensions; (6) Calculate the acceleration and velocity; (7) Update the position of the individual; (8) Repeat step (3) to (7) until the termination condition is reached; (9) End.

### Simulated annealing algorithm

The simulated annealing algorithm [32] achieves the optimization of the objective by simulating the process of cooling down the metal after melting. The simulated annealing algorithm has a good local search capability, and the use of the Metropolis criterion as the basis for whether to accept a new solution as expression in Eq (9) has given the simulated annealing algorithm a better ability to jump out of the local optimal solution.


$$P\left\{{}_{e}^{1}\frac{-(E_{t+1}-E_{t})}{KT}\right.\begin{array}{c} E_{t+1}<E_{t}\\ E_{t+1}>E_{t} \end{array}$$ (9)

### Hybrid gravitational search-simulated annealing algorithm

By combining the two algorithms, the hybrid algorithm can have the ability to jump out of the local optimum while having a better global search capability. After combining the simulated annealing algorithm with the gravitational search algorithm, the hybrid algorithm has a better local search capability due to the introduction of the simulated annealing algorithm, but at the same time, it may lead to the generation of a large number of calculations based on low fitness value solutions, which can seriously affect the efficiency of the hybrid algorithm. In order to effectively use the effective information of the elite solutions, improve the efficiency of the hybrid algorithm and speed up the convergence of the algorithm, the individuals in the population are sorted by fitness value after each iteration, and the individuals are searched by simulated annealing based on the information of the elite solutions.

The basic steps of the hybrid algorithm are: (1) determine the search space; (2) randomize the initialized population; (3) calculate the individual fitness values; (4) rank all individuals in the population to determine the elite solutions (5) perform local search on individuals according to the simulated annealing algorithm (6) update G(t), best(t), worst(t) and; (7) calculate the joint forces in different dimensions; (8) calculate the acceleration and velocity; (9) update the position of individuals; (10) repeat steps (3) to (9) until the termination condition is satisfied; and (11) end.

### Algorithm testing

**Fig 1:**
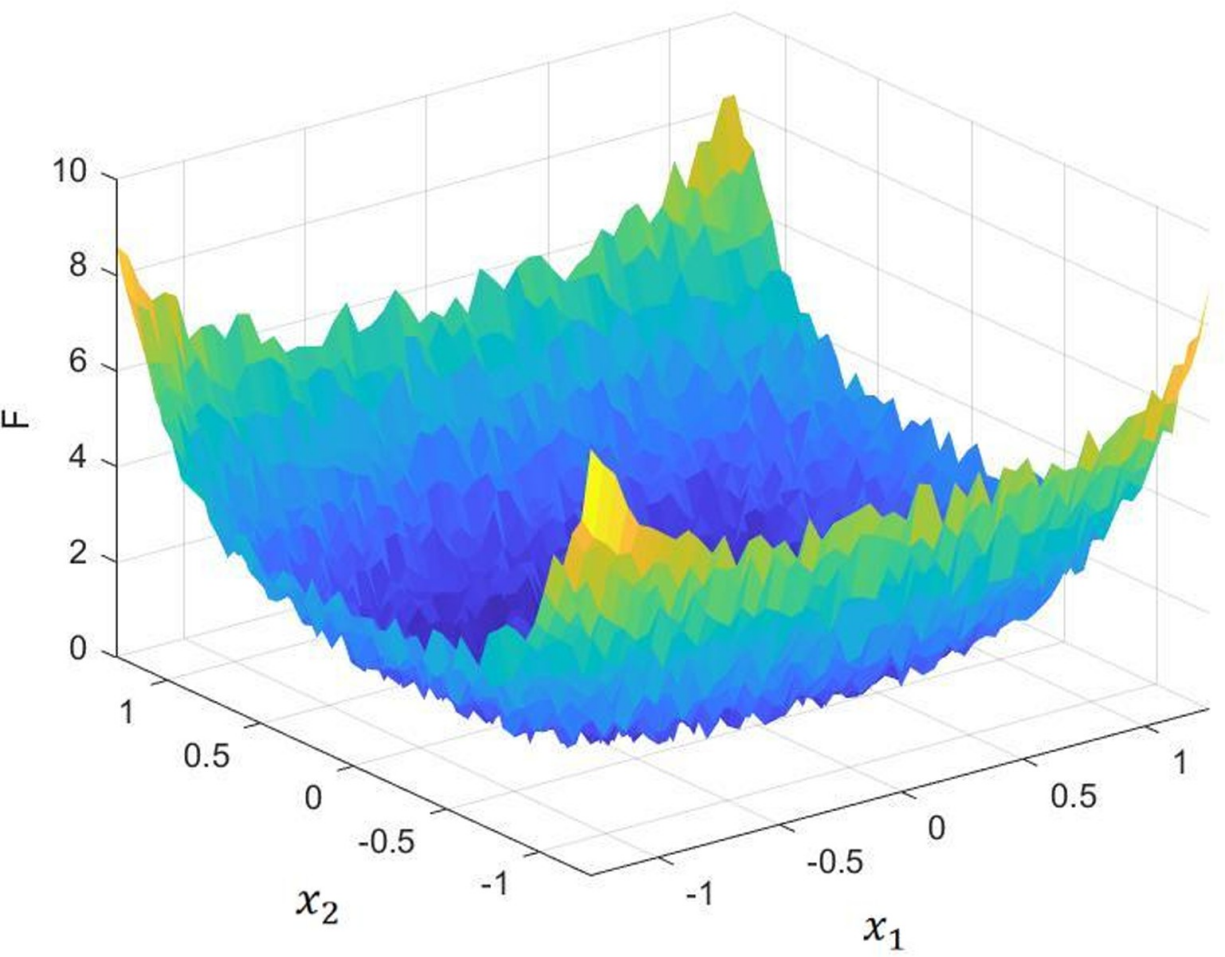
Test function.

**Fig 2:**
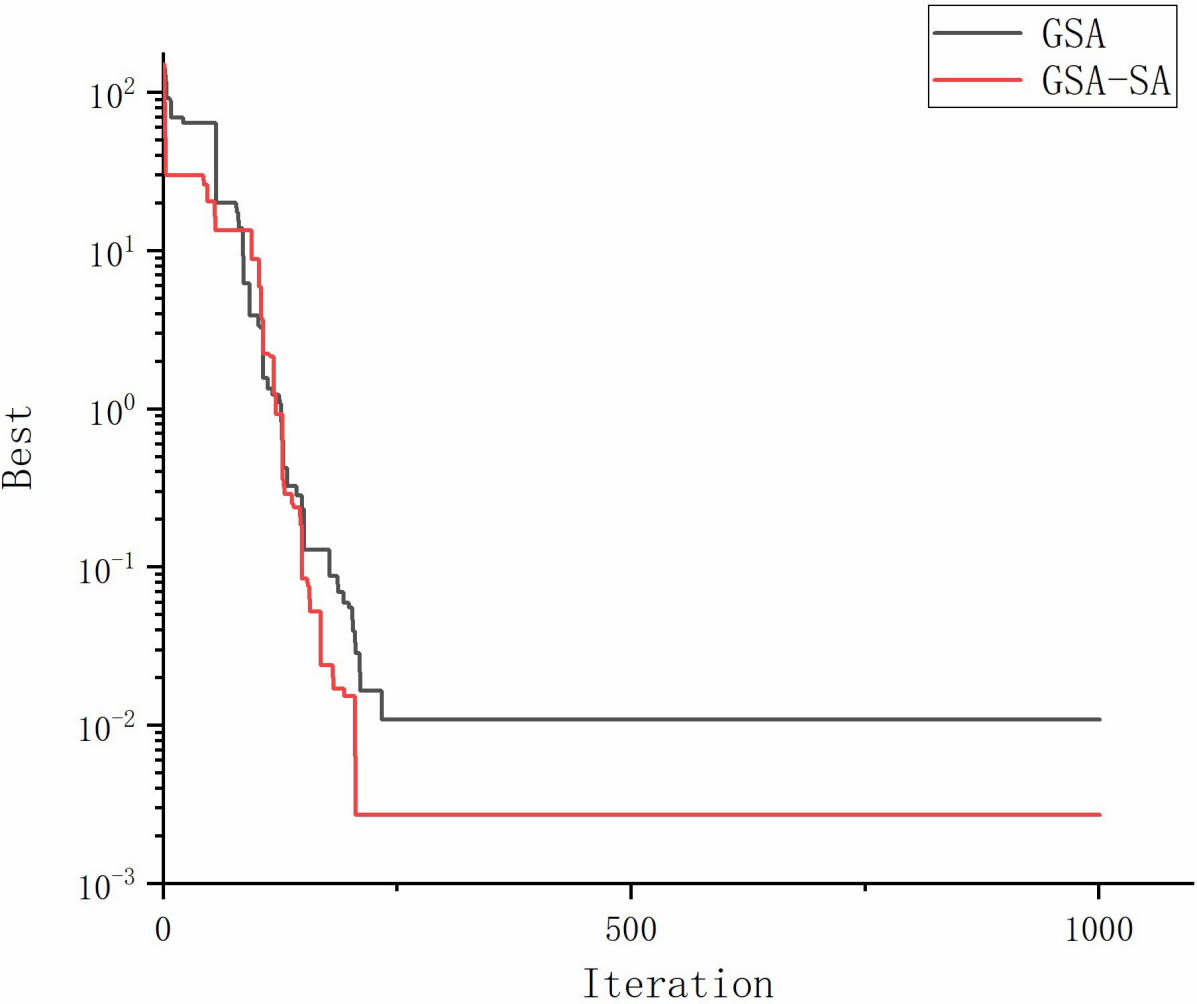
Test function comparison result.


A trial calculation of the hybrid algorithm using single-peak/multi-peak/fixed-dimensional multi-peak test functions such as Eq (10). The image of Eq (10) is shown in Fig 1 and the results are shown in Fig 2.

$$F(x)=\sum_{i=1}^{n}ix^{4}+random[0,1)$$ (10)

Comparing the optimization results, we can see that the hybrid algorithm has some improvement in solution accuracy and convergence speed compared with the original algorithm.

## Gear dynamic model and optimization parameters

### Spur gear dynamic model

**Fig 3:**
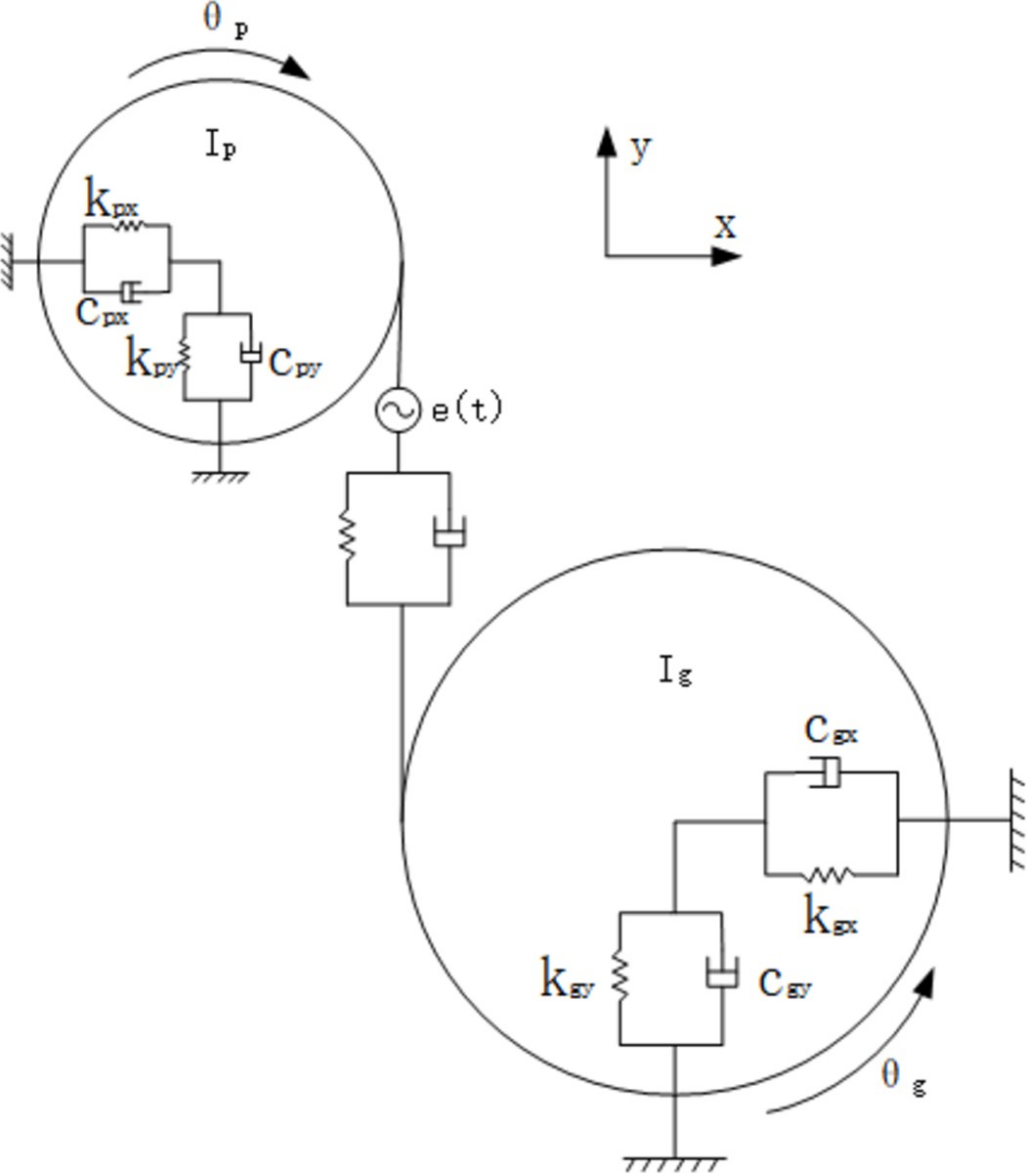
Dynamic model.

In order to analyze the vibration response of the gear pair, a vibration analysis model has been established. According to the gear dynamics theory, a six-degree-of-freedom meshing coupling type vibration analysis model is established as shown in Fig 3 [33–35].

The dynamics equations are:

$$\begin{array}{l} m_{p}{\ddot{x}}_{p}+c_{p}{\dot{x}}_{p}+k_{p}x_{p}=F_{f}\\ m_{p}{\ddot{y}}_{p}+c_{p}{\dot{y}}_{p}+k_{p}y_{p}=-F\\ I_{p}\theta_{p}=T_{p}-F_{p}r_{p}+T_{f1}\\ m_{g}{\ddot{x}}_{g}+c_{g}{\dot{x}}_{g}+k_{g}x_{g}=-F_{f}\\ m_{g}{\ddot{y}}_{g}+c_{g}{\dot{y}}_{g}+k_{g}y_{g}=F\\ I_{g}\theta_{g}=-T_{g}+F_{g}r_{g}+T_{f2} \end{array}$$ (11)

where *m*_*p*_ , *m*_*g*_ are the masses of the wheel and pinion

*y*_*p*_,*y*_*g*_ are the vibration displacement of t the wheel and pinion

*I*_*p*_,*I*_*g*_ are the rotational inertia of the wheel and pinion

where F is the meshing force of the gear pair:

$$F=k_{n}\delta +c_{n}\dot{\delta }$$ (12)

where *δ* is the relative displacement of the gear mesh pair in the direction of the mesh line due to vibration and error:

$$\delta =y_{p}-y_{g}+r_{p}\theta_{p}-r_{g}\theta_{g}-e(t)$$ (13)

where *r*_*p*_, *r*_*g*_ are the radius of the wheel and pinion meshing point

*θ*_*p*_, *θ*_*g*_ are the wheel and pinion vibration angular displacement value

*e*(*t*) is the transmission error of the gear pair

### Addendum modification coefficient and gear profile modification parameters of spur gears

**Fig 4:**
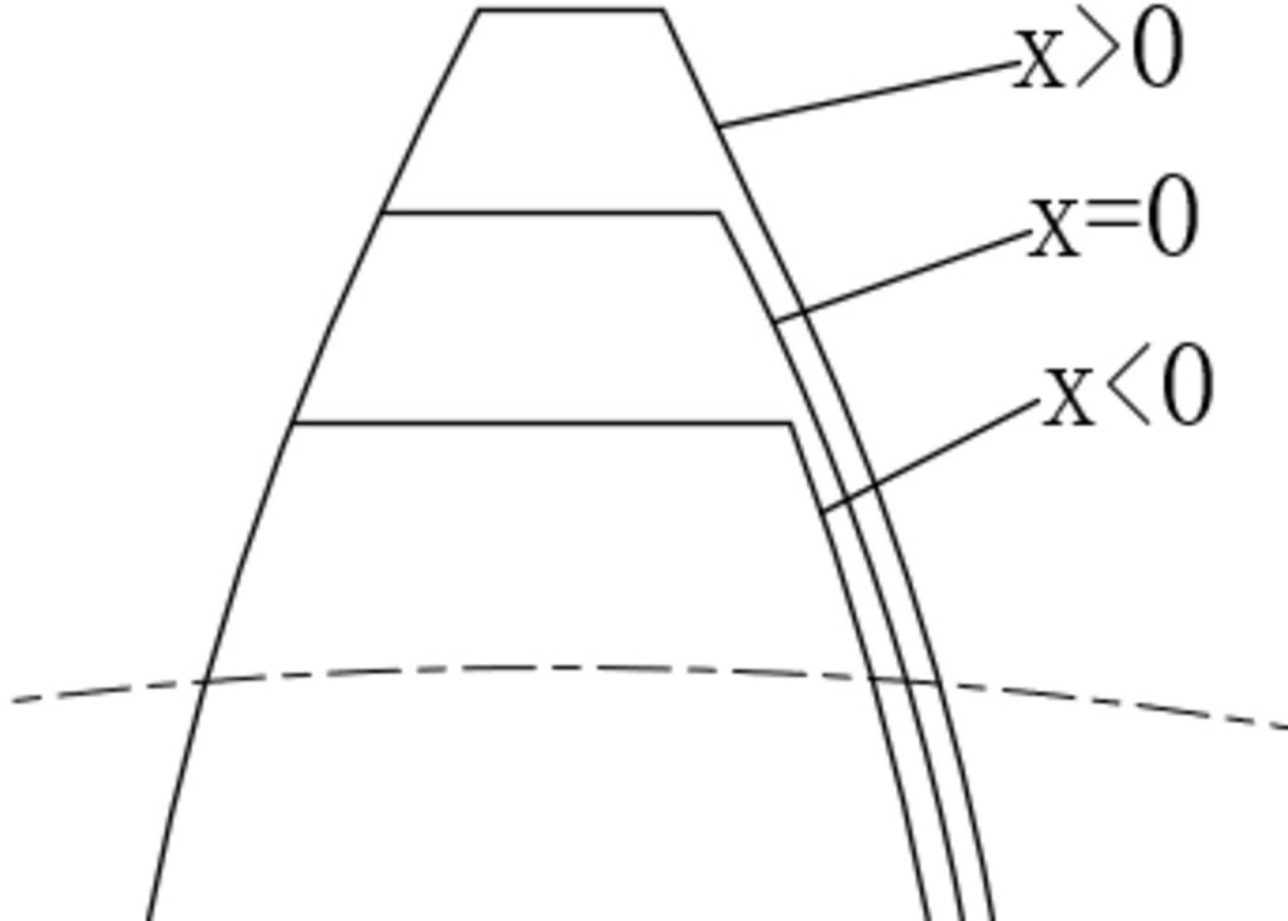
Gear addendum modification coefficient.

There are many methods to select the gear addendum modification coefficient, in order to optimize the solution with the displacement coefficient as the optimization variable, this paper takes the gear design specification as the restriction condition to find the value range of the gear addendum modification coefficient. The effect of gear modification on tooth profile is shown in Fig 4.

To ensure the reliability of the gear, the following constraints are used as constraints: (1) no heel cut during meshing; (2) no top cut during meshing; (3) tooth top thickness greater than 0.4 module; (4) the contact ratio greater than 1.2; (5) no transition interference curve.

**Fig 5:**
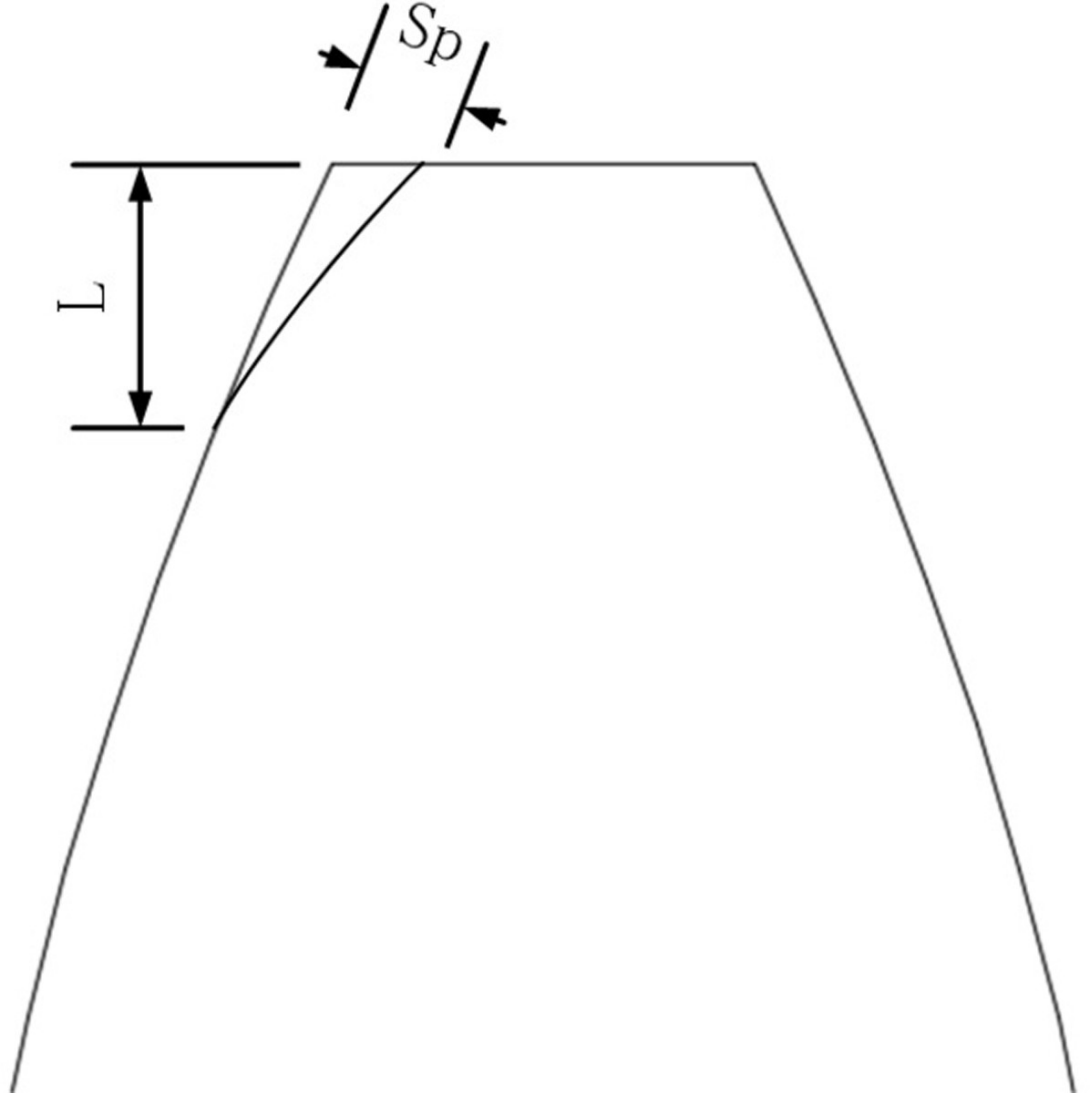
Gear profile modification.

Due to the gear deformation under load and manufacturing errors, meshing interference and impact are prone to occur. Gear profile modification can reduce meshing impact and meshing interference, ensuring smooth operation of the gear. Gear profile modification is to cut off part of the material on the top and root of the tooth to change the shape of the tooth profile as shown in Fig 5. There are various options for gear profile modification depending on the modification position and modifying a single gear or gear pair at the same time. In this paper, only the pinion tooth top is modified. The main factors affecting the profile modification are the modification curve, the modification length, and the maximum modification volume. There are generally linear and curvilinear shaping curves, and this paper uses the most commonly used secondary parabolic profile modification. The maximum trim amount of the gear is generally taken as 0.02module [36].

**Fig 6:**
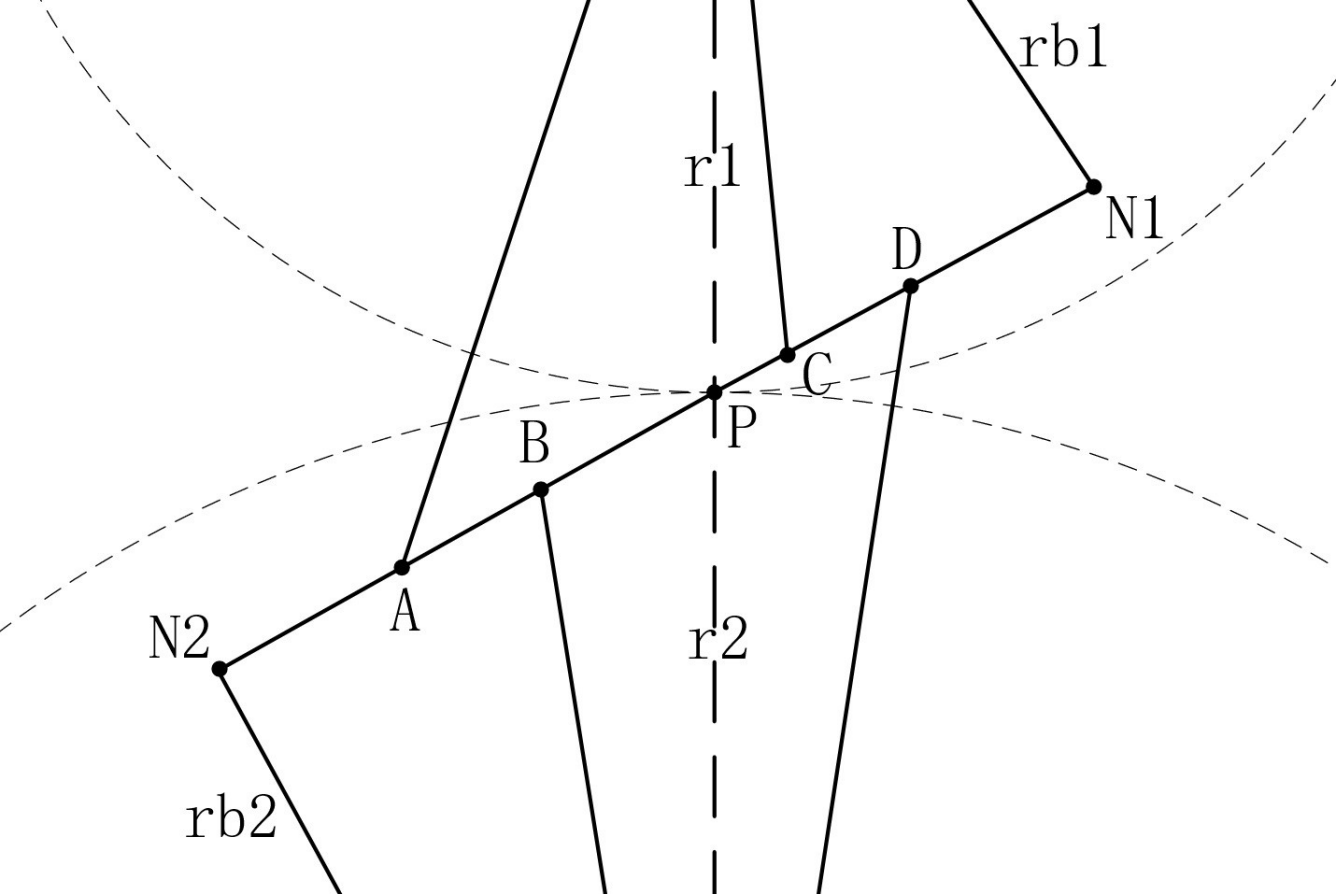
Gear meshing line.


In Fig 6, points B and C are the division points of single and double tooth meshing area, and the position projected from points B and C to the involute as the starting point of profile modification is long profile modification, and the position projected from the midpoint of AB and CD to the involute as the starting point of profile modification is short profile modification. In order to find the optimal modification length, the long modification is chosen as the upper limit of the range of the modification length variable, and its value is determined according to Eq (14).

$$L=(\varepsilon -1)p_{b}$$ (14)

where *ε* is the contact ratio of the gear pair, *p*_*b*_ is the base circle tooth pitch

## Example calculation and analysis

### The range of variable values

The optimization objective function is established with the vibration response of the gears shown in Table 1, and the optimal addendum modification coefficient and profile modification parameters are searched by the hybrid gravitational search-simulated annealing algorithm.

**Table 1:** Gear parameters.

Gear parameters	Wheel	Pinion
Number of teeth	45	36
Module	1	1
Pressure angle	20°	20°
Helix angle	0	0
Face width	8mm	7mm
Addendum coefficient	1	1
Tip gap coefficient	0.25	0.25

The gears are addendum modifications and the profile modification is a quadratic parabolic modification with only the pinion tooth tops trimmed. Calculate the range of values of the variables with the method in Addendum modification coefficient and gear profile modification parameters of spur gears section. The results are shown in Table 2.

**Table 2:** The range of values of the variables.

Variables	Value range
addendum modification coefficient of wheel (*x*_1_)	(-1,0]
addendum modification coefficient of pinion (*x*_2_)	[0,1)
Amount of profile modification (*S*_*p*_)	(0,0.02]
Length of profile modification (L)	[0.2108,2.1083]

### Objective function

In order to effectively evaluate the optimization results, dynamic load coefficient, root mean square(RMS) value of meshing force fluctuation amplitude (*N*), RMS value of pinion vibration torsional acceleration (*rad* / *s*^2^), and RMS value of pinion vibration displacement acceleration (*m* / *s*^2^) are selected as evaluation indicators, the meshing force fluctuation amplitude is the difference between the maximum and minimum values of meshing force.

The optimization objective function is obtained by combining the weighting by G1-variance coefficient method. The G1 method is a highly subjective weighting method, in order to better reflect the vibration response of gears, the variation coefficient method is introduced to combine weights. The variation coefficient is an objective weighting method, and the combination of the two methods can better reflect the vibration response of gears.

The G1 method first ranks the evaluation indicators, then compares the relative importance between the indicators Relative importance and determines the importance ratio by the relative importance assignment table shown in Table 3, and calculates the G1 method weights by Eqs (15) and (16) according to the relative importance ratio.

**Table 3:** Relative importance assignment table.

Scale	Meaning
1.0	Equal importance
1.2	Slightly important
1.4	Obviously important
1.6	very important
1.8	extremely important


$$\omega_{m}=(1+\sum_{k=2}^{m}\overset{m}{\underset{i=k}{∐}}r_{i}{)}^{-1}$$ (15)

$$\omega_{k-1}=r_{i}\omega_{k},k=m,m-1,\ldots ,3,2$$ (16)

The evaluation indexes are ranked in order of importance from highest to lowest: dynamic load coefficient > meshing force fluctuation amplitude > pinion vibration displacement acceleration > pinion vibration torsion acceleration.

Referring to Table 3 can get the importance ratio of *r*_2_ = 1.2, *r*_3_ = 1.1, *r*_4_ = 1.1. The weight of the G1 method can be calculated by substituting into Eqs (15) and (16).


$$\omega_{m}=\left\{0.305,0.254,0.231,0.210\right\}$$

The coefficient of variation method is an objective weighting method that calculates the weights by processing the data of the evaluation indexes. The coefficient of variation method first calculates the mean and standard deviation of evaluation indexes from Eqs (17) and (18).


$$\bar{x}=\frac{1}{n}\sum_{i=1}^{n}x_{i}$$ (17)

$$\sigma =\sqrt{\frac{1}{n}\sum_{i=1}^{n}{\left(x_{i}-\bar{x}\right)}^{2}}$$ (18)

Then the coefficient of variation V of the evaluation index is calculated as:

$$V=\frac{\sigma }{\bar{x}}$$ (19)

Finally, the coefficient of variation is normalized to obtain the weight of the variation coefficient method as shown in Eq (20).


$$\omega_{n}=\frac{V}{\sum_{i=1}^{n}V_{i}}$$ (20)

Substituting the vibration response under different working conditions into Eqs (16)–(18) yields the coefficient of variation

$$V=\left\{4\times 10^{-5},0.191,1.9307,0.1148\right\}$$

Normalization yields:

$$\omega_{n}=\left\{1.789\times 10^{-5},0.085,0.863,0.051\right\}$$

Let the combination weights be

$$\omega =k\omega_{m}+(1-k)\omega_{n}$$ (21)

The value of k is minimally determined by solving the squared sum of the deviations of the combination weights from the G1 method and the coefficient of variation method, and the combination weights are calculated by substituting the weights of the G1 and coefficient of variation methods as

$$\omega =\left\{0.1525,0.1695,0.5470,0.1305\right\}$$

### Optimization result

#### Results of only optimizing modification coefficients or profile modification parameters

**Fig 7:**
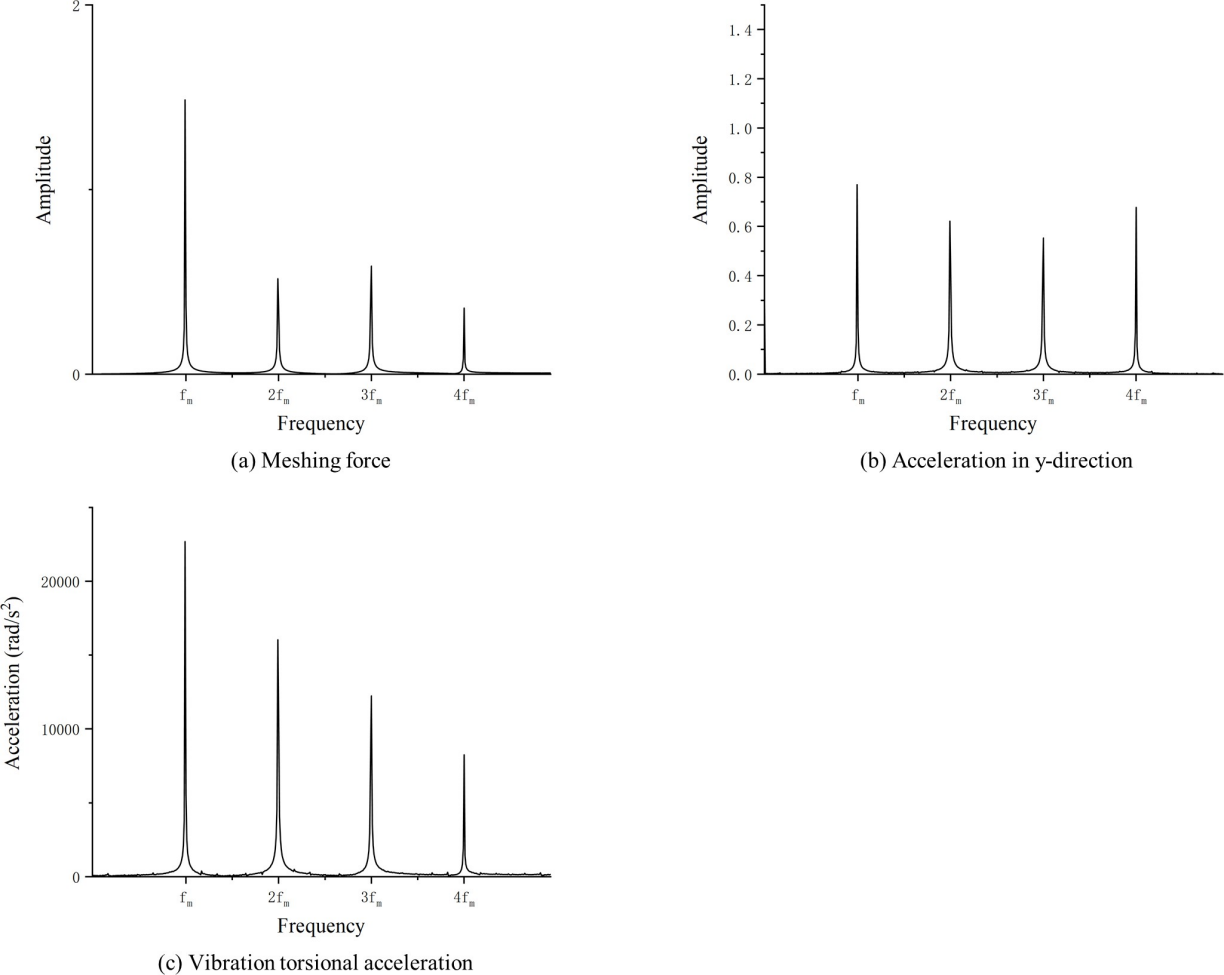
Optimization results of modification coefficient(Frequency).

**Fig 8:**
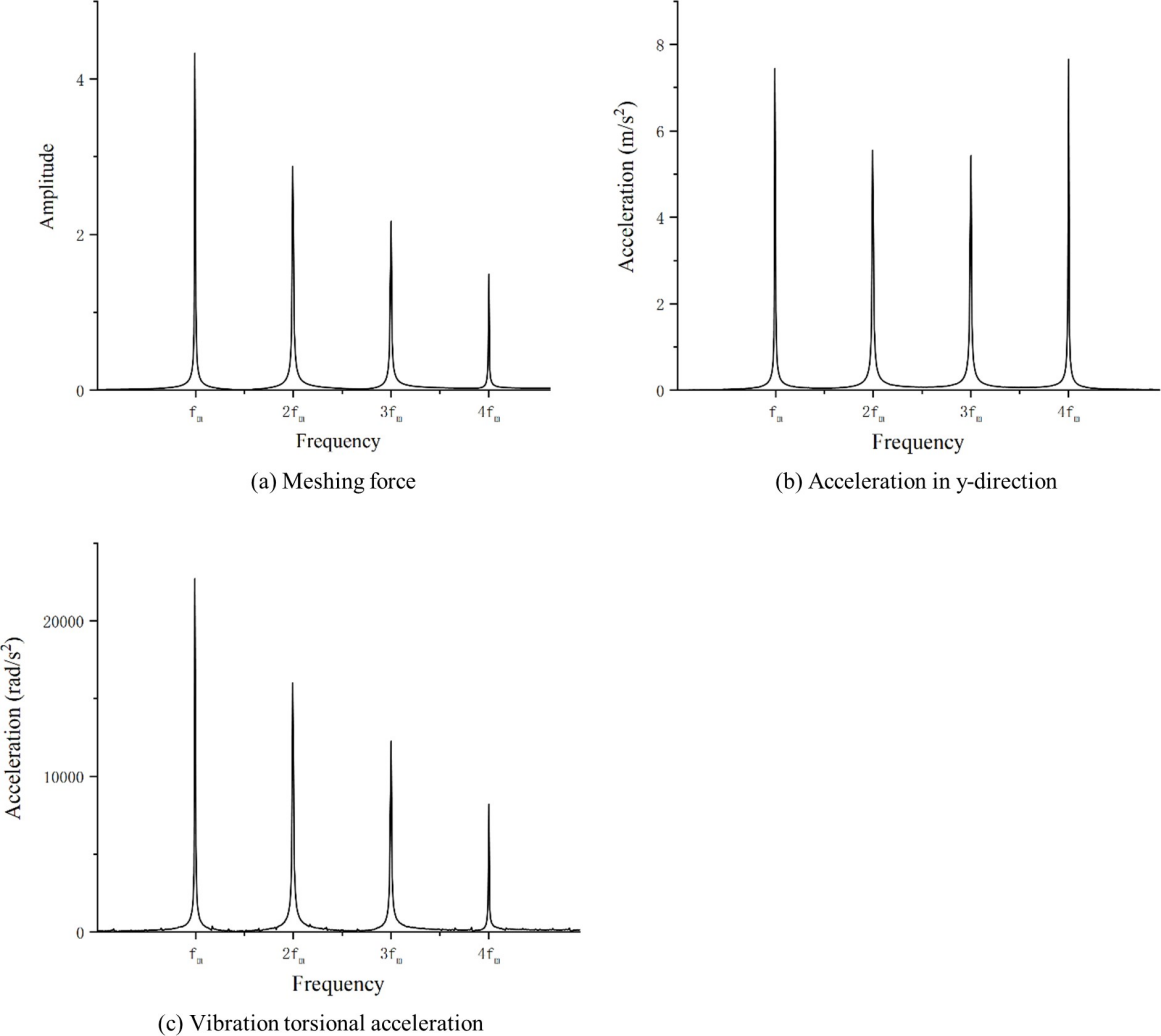
Optimization results of profile modification(Frequency).

The results of optimizing the addendum modification coefficients and profile modification parameters for the gears shown in Table 1, respectively, are shown in Table 4, and the vibration response of the optimized gears is processed to obtain the frequency domain plots shown in Figs 7 and 8.

**Table 4:** Single optimization result.

Parameters	modification coefficients	profile modification
*x*_1_	-0.4994	
*x*_2_	0.4994	
*S*_*p*_		0.0152
L		1.4239

As shown in Tables 5 and 6, the vibration response of the gear pair is also improved by only optimizing the addendum modification coefficients or profile modification parameters, which is 34.7% and 13.2% lower than that before optimization, respectively. The non-optimized gear vibration response is shown in Fig 10. Therefore, the vibration suppression effect of both modification coefficients or profile modification optimization is better than that of only modification coefficients or profile modification optimization of gears.

**Table 5:** Result of modification coefficients optimization.

Variables	Value range
dynamic load coefficient	1
meshing force fluctuation amplitude	14.17
pinion vibration displacement acceleration	62.26
pinion vibration torsional acceleration	3.35◊10^4^

**Table 6:** Result of profile modification optimization.

Variables	Value range
dynamic load coefficient	1
meshing force fluctuation amplitude	30.52
pinion vibration displacement acceleration	73.19
pinion vibration torsional acceleration	3.36◊10^4^

#### Results of optimizing both modification coefficients or profile modification parameters

**Fig 9:**
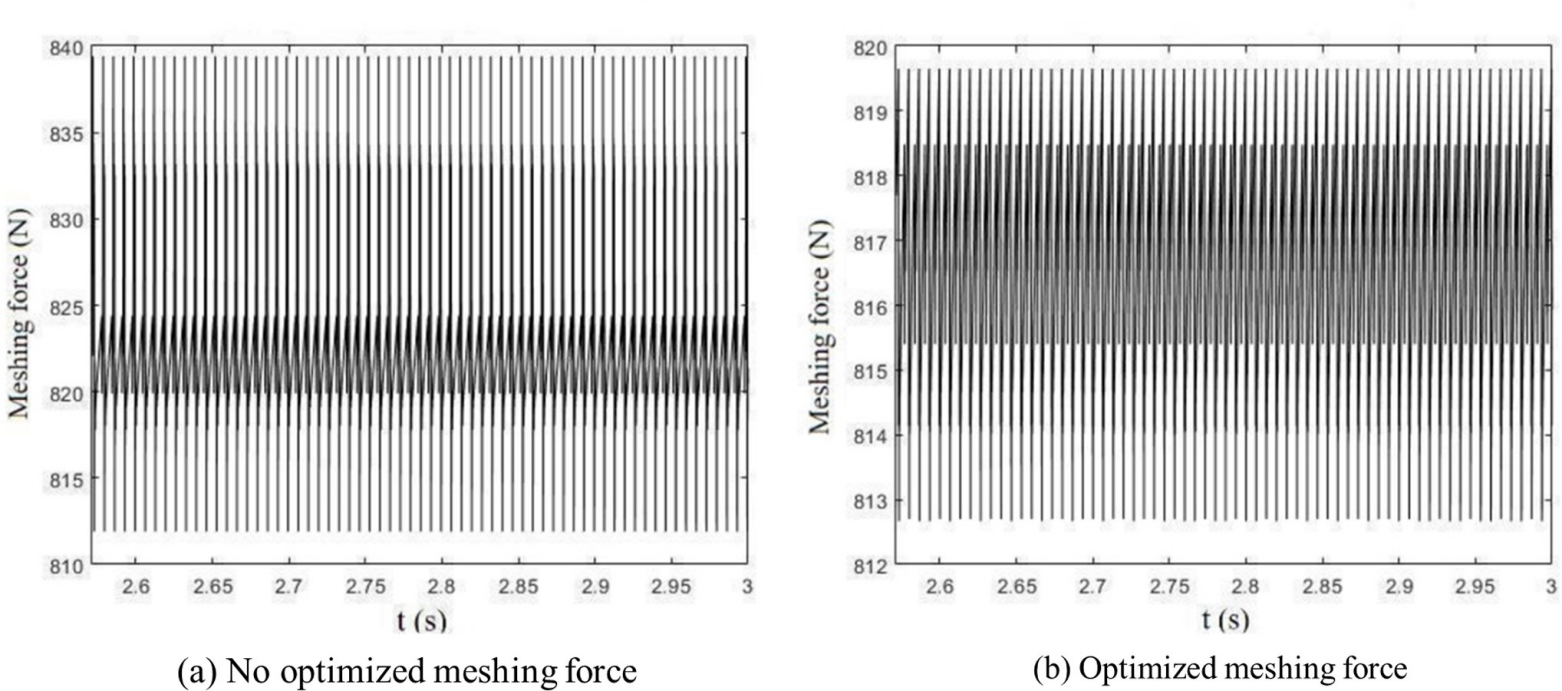
Meshing force.

The addendum modification coefficients and profile modification parameters optimized by the GSA-SA hybrid algorithm are shown in Table 7. Fig 9 shows the time domain diagram of the optimized gear meshing force and meshing force without optimization.

**Table 7:** Optimization result.

Parameters	Result
*x*_1_	-0.9187
*x*_2_	0.9187
*S*_*p*_	0.0199
L	1.2881

As shown in Table 8 the vibration response of the optimized gears was reduced by 40.6% overall compared with that before optimization, including a 65.9% reduction in the amplitude of meshing force fluctuation, a 45% reduction in pinion vibration displacement acceleration, and a 0.3% reduction in pinion vibration displacement acceleration.

**Table 8:** Result comparison.

Variables	Optimized	No optimized
dynamic load coefficient	1	1
meshing force fluctuation amplitude	12.84	40.28
pinion vibration displacement acceleration	46.30	84.16
pinion vibration torsional acceleration	3.37◊10^4^	3.38◊10^4^

**Fig 10:**
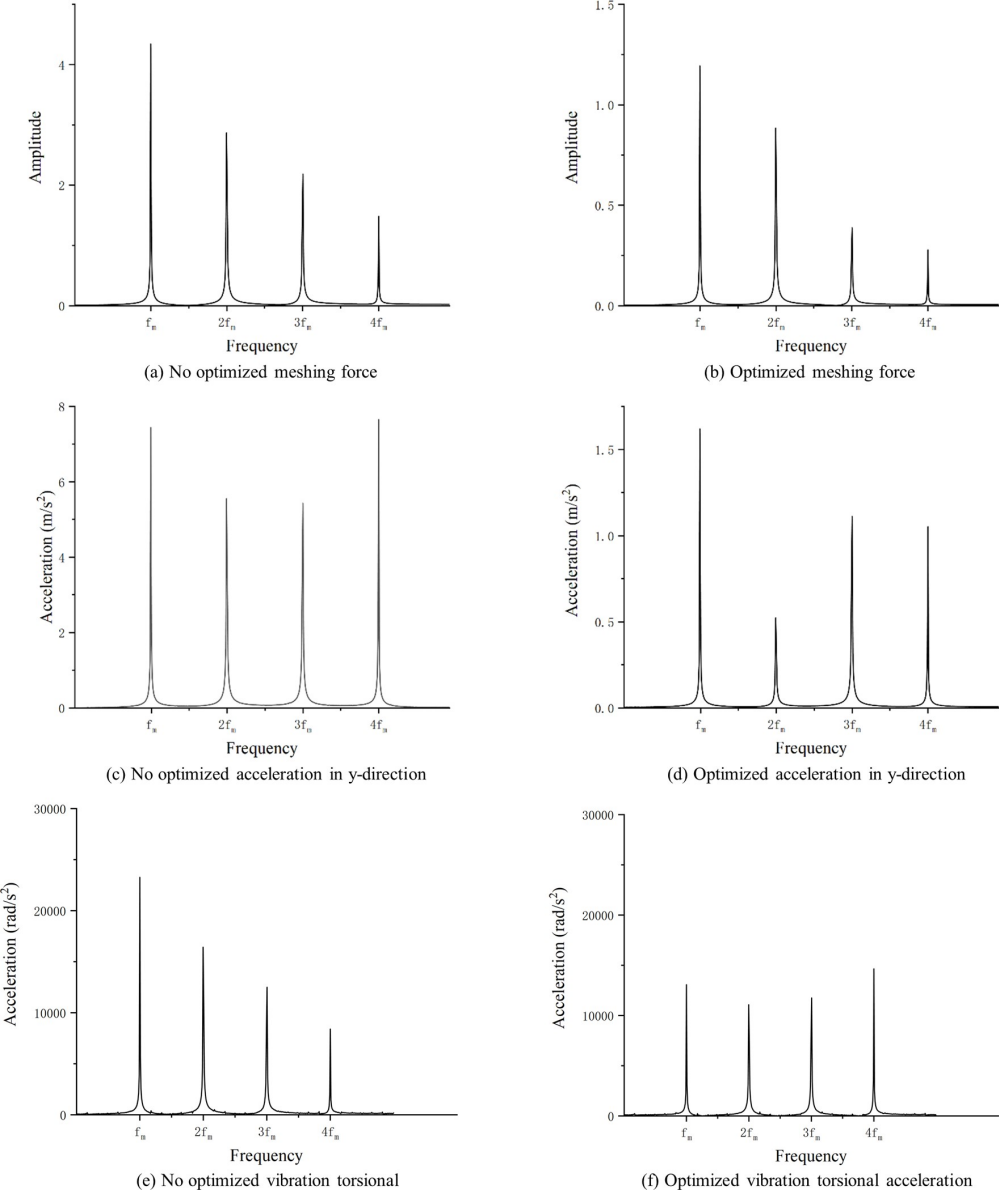
Comparison of optimization results(Frequency).

The optimization results are analyzed in the frequency domain and the frequency domain plots are shown in Fig 10.

As shown in Fig 10, the gear meshing force is mainly affected by the mesh frequency which reduced from 4.3 to 1.2, and the first four harmonics are all reduced after optimization. y-direction vibration acceleration is also reduced after optimization, and the greatest reduction is at the second harmonic, reduced from 5.5 to 0.6. The torsional acceleration decreases at the GMF and second harmonic and increases at the third harmonic and fourth harmonic.

## Conclusion

(1) A gravity search-simulated annealing hybrid algorithm is proposed based on the gravity search algorithm. By combining the simulated annealing algorithm, the hybrid algorithm improves the ability of the gravity search algorithm to jump out of local optima to a certain extent. The feasibility of the hybrid algorithm was verified through comparative analysis using test functions for trial calculations.
(2) Based on the six-degree-of-freedom gear dynamic model and GSA-SA hybrid algorithm proposes a method for optimizing the design parameters of spur gears. Optimize the gear addendum modification coefficient and profile modification parameters through heuristic algorithm to reduce the level of vibration and noise of the gear pair.
(3) Through the analysis of the results of the optimization case, it can be seen that the vibration reduction effect of optimizing the addendum modification coefficient and profile modification parameters of the gear at the same time is better than that of only optimizing the addendum modification coefficient or profile modification parameters.
(4) This paper studies the vibration optimization method of gear pairs based on gear dynamics and heuristic algorithm. On this basis, further research can be carried out on the optimization methods for the vibration and noise reduction of complex gear transmission systems, providing reference for the vibration and noise reduction optimization in engineering.

## Supporting information

S1 Data Relevant data. (XLSX)
